# Haemoptysis in Pulmonary Arterial Hypertension Associated with Congenital Heart Disease: Insights on Pathophysiology, Diagnosis and Management

**DOI:** 10.3390/jcm11030633

**Published:** 2022-01-26

**Authors:** Amalia Baroutidou, Alexandra Arvanitaki, Adam Hatzidakis, Georgia Pitsiou, Antonios Ziakas, Haralambos Karvounis, George Giannakoulas

**Affiliations:** 1 First Department of Cardiology, AHEPA University Hospital, Aristotle University of Thessaloniki, St. Kiriakidi 1, 54636 Thessaloniki, Greece; bamalia27@gmail.com (A.B.); alexandra.arvanit@gmail.com (A.A.); adamhatz@hotmail.com (A.H.); aziakas@auth.gr (A.Z.); hkarvounis@auth.gr (H.K.); 2 Adult Congenital Heart Centre, National Centre for Pulmonary Arterial Hypertension, Royal Brompton Hospital, Guy’s and St Thomas’ NHS Foundation Trust, Sydney Street, London SW3 6NP, UK; 3 Department of Respiratory Medicine, G. Papanikolaou Hospital, 57010 Thessaloniki, Greece; gpitsiou@yahoo.gr

**Keywords:** haemoptysis, congenital heart disease, pulmonary arterial hypertension, Eisenmenger syndrome

## Abstract

Haemoptysis represents one of the most severe major bleeding manifestations in the clinical course of pulmonary arterial hypertension (PAH) associated with congenital heart disease (CHD). Accumulating evidence indicates that dysfunction of the pulmonary vascular bed in the setting of PAH predisposes patients to increased hemorrhagic diathesis, resulting in mild to massive and life-threatening episodes of haemoptysis. Despite major advances in PAH targeted treatment strategies, haemoptysis is still correlated with substantial morbidity and impaired quality of life, requiring a multidisciplinary approach by adult CHD experts in tertiary centres. Technological innovations in the field of diagnostic and interventional radiology enabled the application of bronchial artery embolization (BAE), a valuable tool to efficiently control haemoptysis in modern clinical practice. However, bleeding recurrences are still prevalent, implying that the optimum management of haemoptysis and its implications remain obscure. Moreover, regarding the use of oral anticoagulation in patients with haemoptysis, current guidelines do not provide a clear therapeutic strategy due to the lack of evidence. This review aims to discuss the main pathophysiological mechanisms of haemoptysis in PAH-CHD, present the clinical spectrum and the available diagnostic tools, summarize current therapeutic challenges, and propose directions for future research in this group of patients.

## 1. Introduction

Pulmonary arterial hypertension (PAH) is a rare, heterogeneous disease of the pulmonary vasculature, haemodynamically defined by a mean pulmonary arterial pressure (mPAP) >20 mmHg, a normal pulmonary artery wedge pressure ≤15 mmHg and elevated pulmonary vascular resistance ≥3 Wood units [1]. Congenital heart disease (CHD) is frequently complicated by PAH, including four individual groups with shared features; Eisenmenger syndrome (ES), congenital systemic to pulmonary shunts, PAH associated with coincidental or small defects, and PAH encountered in patients with repaired congenital defects [2]. Although the use of PAH targeted pharmacotherapy has substantially improved functional capacity and survival in these patients over the last years, the morbidity burden remains high, affecting their quality of life and posing an impact on healthcare systems globally [3,4].

Spontaneous bleeding events are common in PAH-CHD and usually minor and self-limiting (e.g., dental bleeding, epistaxis, easy bruising, menorrhagia). Haemoptysis is one of the most perilous major bleeding manifestations in the clinical course of PAH-CHD and can be life-threatening. Its incidence is estimated as 3.1% to 5.5% [5,6] in PAH-CHD patients, whereas it is markedly elevated in patients with ES, accounting for 6% to 49% of cases [7,8,9,10,11,12]. Although it is an alarming symptom for patients and physicians, haemoptysis seems to be a relatively infrequent cause of death, accounting only for 3% of deaths in a recent international, multi-centre, retrospective study of patients with ES [12]. Haemoptysis requires instant management and constitutes a major clinical diagnostic and therapeutic challenge. Technological advances of modern medicine led to the application of bronchial artery embolization (BAE), a minimally invasive technique for managing massive and recurrent haemoptysis [13,14], raising hopes for relapse prevention [15]. Furthermore, little evidence is available on the impact and management of anticoagulation therapy either for primary or secondary prevention in patients with concurrent haemoptysis, constituting an inextricable clinical dilemma.

The current review exhibits the pathophysiology and clinical manifestations of haemoptysis in patients with PAH-CHD, presents current diagnostic strategies, discusses therapeutic management options and highlights areas of uncertainty and gaps in current evidence.

## 2. Pathophysiology of Haemoptysis

**Figure 1:**
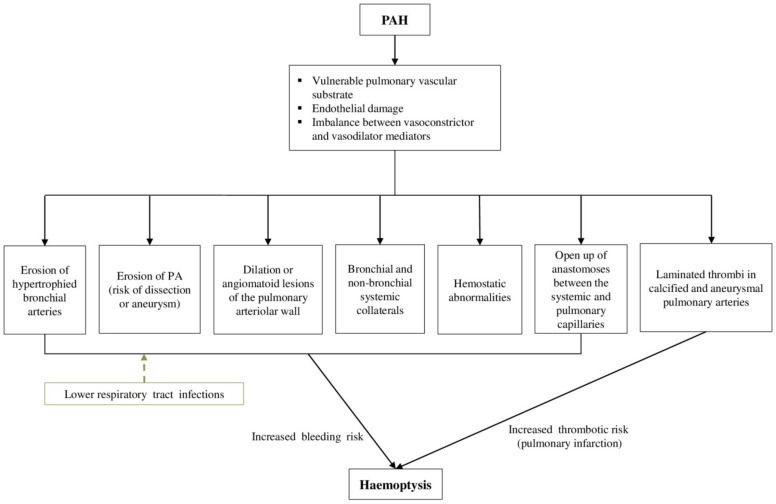
Pathophysiology of haemoptysis in patients with PAH-CHD. CHD: Congenital heart disease, PAH: Pulmonary arterial hypertension, PA: Pulmonary artery.

The underlying pathophysiology of haemoptysis in PAH-CHD remains intricate and involves various, complex mechanisms (Figure 1). Obliterative remodeling of the pulmonary vascular bed, pulmonary microvascular changes, endothelial damage, vasoconstriction and thrombosis have been correlated with the progression of PAH [16,17,18,19].

All these processes lead to a vulnerable pulmonary vascular substrate in PAH-CHD, in which episodes of haemoptysis may occur, especially after shunt reversal and the establishment of fixed pulmonary vascular disease. Robust data about the exact mechanisms of haemoptysis are lacking. The erosion of hypertrophied bronchial arteries into a bronchus has been described as a possible explanation, but the reason for this bronchial arterial hypertrophy remains unidentified [20]. The high-pressure bronchial circulation is the primary source of bleeding in the majority of massive haemoptysis cases (90%) [21]. Nonetheless, not all cases of haemoptysis can be attributed to eroded bronchial arteries. In approximately 5% of the cases, bleeding originates from the pulmonary vessels [22,23]. Existing pulmonary artery (PA) dissection or aneurysm are vulnerable to rupture, in the setting of increased PAP in PAH, and, in several cases, they have been associated with haemoptysis [20,24]. Furthermore, dilation or angiomatoid lesions of the pulmonary arteriolar wall are frequently encountered in patients with PAH-CHD (mainly with large post-tricuspid shunts) due to the presence of pulmonary vascular obstructive disease, and have been related to haemoptysis [20].

Furthermore, the release of angiogenic growth factors, triggered by the inflammatory response in PAH-CHD, induces neoangiogenesis and the development of bronchial and non-bronchial systemic collateral circulation [13,25,26]. Systemic aortopulmonary collateral vessels may emerge from the subclavian, intercostal, thoracic branches of the axillary artery, internal mammary arteries and infradiaphragmatic branches from the inferior phrenic, left gastric, and celiac axis [13]. These newly formed collaterals are displayed to high systemic arterial pressures and therefore are sensitive to dilation and rupture, increasing the risk of haemoptysis [13,25].

The pulmonary arteries account for 99% of the arterial blood supply to the lungs and take part in gas exchange, while the bronchial arteries provide nourishment to the supporting structures of the airways. The bronchial vasculature is in close proximity to the pulmonary arteries at the level of the vasa vasorum where the two systems are connected by thin-walled anastomoses between the systemic and pulmonary capillaries. These anastomoses may open up in regions of the lung that are deprived of pulmonary arterial blood flow due to pulmonary vascular obstructive disorders in the setting of PAH-CHD. Consequently, these fragile vessels are subjected to increased systemic arterial pressure and can lead to haemoptysis by rupturing into the alveoli or bronchial airways [27,28]. Finally, in a minority of cases (5%), massive haemoptysis may arise from the aorta (aortobronchial fistula, ruptured aortic aneurysm) or the nonbronchial systemic circulation [21].

Abnormalities of the hemostatic mechanisms in patients with ES could also explain the hemorrhagic diathesis in these patients. Hemostatic abnormalities are attributed both to platelet disorders (thrombocytopenia and thrombasthenia) and abnormalities in coagulation pathways (overactivation), while increased hematocrit has been correlated with impaired fibrinogen function [29,30,31]. Vitamin K-dependent clotting factors (prothrombin, factors VII and IX) and factor V are reduced due to hypoxic induced impaired liver synthesis, fibrinolytic activity is increased, and the largest von Willebrand multimers are depleted [29]. Additionally, right-to-left shunting in ES delivers megakaryocytes into the systematic circulation, bypassing the lungs where megakaryocytic cytoplasm is normally fragmented into platelets, and thus is associated with thrombocytopenia [32]. Furthermore, prostanoid use has been associated with the inhibition of platelet function that could trigger haemoptysis [33]. Recently, another prospective open-label study of riociguat in patients with PAH reported haemoptysis in 2.5% of patients (n = 8). Of the six patients with serious hemoptysis, three were receiving concomitant anticoagulants, two were receiving a concomitant prostanoid, and one was receiving concomitant antiplatelet therapy [34]. Therefore, the role of PAH targeted therapies in triggering haemoptysis and the underlying mechanisms have yet to be proved.

On the other hand, patients with PAH-CHD are at increased risk for thromboembolic events that could equally contribute to the occurrence of haemoptysis. Thrombosis is associated with blood stasis in dilated heart chambers and pulmonary arteries, atherosclerosis and/or endothelial dysfunction, atrial arrhythmias and the presence of thrombogenic material (e.g., conduits). Laminated thrombi in large, partially calcified and aneurysmal pulmonary arteries are common, may occur in up to 30% of patients with ES [10], and have been associated with pulmonary infarction that could be related with massive haemoptysis as pointed out in the Paul Wood series [35].

Acute lower respiratory tract infections could be an additional aggravating factor in patients with PAH-CHD. Inflammation of the tracheobronchial tree renders airways congested, friable, and therefore susceptible to bleeding. Chronic respiratory tract infections lead to an increase in systemic arterial flow via formation of new vessels, which in turn are prone to rupture and cause haemoptysis [27].

## 3. Clinical Manifestations

There is a marked wide variability in the spectrum of haemoptysis, ranging from mild episodes to massive ones that potentially lead to acute respiratory failure and hemodynamic instability. Recurrent and “of increasing volume” episodes appear to be common in this population and imply the onset of a downward trend, which sometimes may prove fatal [35,36,37].

Massive haemoptysis, also referred as “major” or “severe” haemoptysis in the literature, describes a large volume of expectorated blood within 24 h. There is no unanimity on the definition of massive haemoptysis in previous studies [38], and cut off values ranging from 100 to more than 1000 mL/24 h have been reported over the years [39,40,41,42]. The aforementioned definitions are all dependent on the amount of bleeding, which in some cases can be over-or underestimated by the patient. Furthermore, the quantity of bleeding is only one parameter that affects the patient’s clinical stability and therefore does not actually reflect the morbidity and mortality [38]. Consequently, it seems to be more important for physicians to estimate each patient’s clinical status individually.

In blood expectoration cases, the differentiation of haemoptysis from haematemesis is highlighted; especially in patients with PAH-CHD and known liver dysfunction or cirrhosis as a result of progressive right heart failure [36,43]. Preceding nausea, dark colored sputum simulating coffee ground in the absence of frothiness, and the presence of food particles compose supporting evidence of hematemesis and require vigilance [44]. Haemoptysis in adults with PAH-CHD is generally accompanied by a deterioration of their overall clinical presentation [45]. Thus, shortness of breath, dyspnea, cough, fatigue and weakness are usually typical concurrent symptoms. Furthermore, on admission, patients presenting with large volume haemoptysis may have signs of progressive right heart failure with peripheral oedema and abdominal distension [46].

In some patients, characteristic adjunct signs and symptoms may emerge, attributable to the specific origin of the vessel, which is responsible for the bleeding. For example, a dilated PA could possibly compress the left recurrent laryngeal nerve, the airway, or the left main coronary artery leading to hoarseness, wheeze or angina, respectively. The rupture or dissection of a PA could also provoke the clinical presentation of cardiac tamponade [2]. Additionally, collateral circulation from bronchial branches of the coronary arteries may lessen the coronary perfusion and cause angina [47]. A high-pitched murmur may be heard under the clavicles, along the sternal borders, and to the side of the vertebrae over the posterior chest due to the blood flow in case of collateral circulation from mammary and intercostal vessels [27].

## 4. Diagnostic Evaluation

The diagnostic approach of haemoptysis in patients with PAH-CHD includes a detailed medical history, clinical examination, laboratory investigations and imaging. Electrocardiography (ECG), although not diagnostic, may provide information on the severity of PH, revealing RV hypertrophy or strain, QTc prolongation and arrhythmias [2]. Blood tests should include total blood count, with an emphasis on haemoglobin concentration), iron studies (ferritin and transferrin saturation), routine biochemistry (liver enzymes, urea and creatinine concentration plasma electrolytes), C-reactive protein levels (to exclude the probability of infection-induced haemoptysis), and international normalized ratio (especially in patients receiving vitamin K antagonists) [2,37].

Chest X-ray, although having a low discriminative ability (47%), may reveal dilated, calcified or aneurysmal central pulmonary arteries [48]. Cardiomegaly, attributable to right chamber enlargement, and consolidations, indicative of pulmonary hemorrhagic features, are also common radiological findings [17,36,49]. Confirmatory of these findings, a high-resolution chest computed tomography (CT) is more helpful to detect pulmonary infarction and parenchymal disease [17]. The basis of the initial evaluation of haemoptysis is computed tomography pulmonary angiography (CTPA). Additional to the dilated pulmonary arterial network, CTPA determines the location of the bleeding and guides potential treatment options. It also enables the depiction of multiple aortopulmonary collateral arteries responsible for haemoptysis and is considered the gold standard to exclude pulmonary embolism [20,37,50].

Transthoracic echocardiography is a non-specific monitoring tool concerning the identification of bleeding in patients with haemoptysis. However, it may prove useful as it provides additional information regarding biventricular size and function, detects the direction of intracardiac shunting [17] and estimates systolic pulmonary arterial pressure (PAP) with the modified Bernoulli equation in the absence of pulmonary valve stenosis [2]. If there is a clinical and echocardiographic suspicion of PAH, patients with CHD and haemoptysis should undergo right heart catheterization for the diagnosis of PAH following bleeding control and respiratory and haemodynamic stabilization as a planned procedure [2].

Cardiac magnetic resonance (CMR) imaging is a further adjunct to evaluate the intracardiac anatomy, the size of the shunt as well as the biventricular function, provided that the patient is hemodynamically stable. Moreover, abnormalities of the pulmonary vascular bed, such as aortopulmonary collaterals, PA aneurysms and in situ pulmonary thrombi may be defined [51]. It also enables an estimation of the pulmonary blood flow and the ventricular volumes [52].

Finally, bronchoscopy constitutes a valuable tool for evaluating and treating haemoptysis in patients with PAH-CHD, as it enables direct access to the location of the pulmonary haemorrhage (identify the culprit vessel) in order to medically or invasively stop the bleeding. It also allows for the receiving of endobronchial biopsies to exclude malignancy in case of suspicion [44,53]. The use of rigid bronchoscopy should be preferred for controlling massive bleeding, especially if the patient is not intubated [54].

## 5. Management of Haemoptysis

### 5.1. Initial Steps and Stabilization

**Figure 2:**
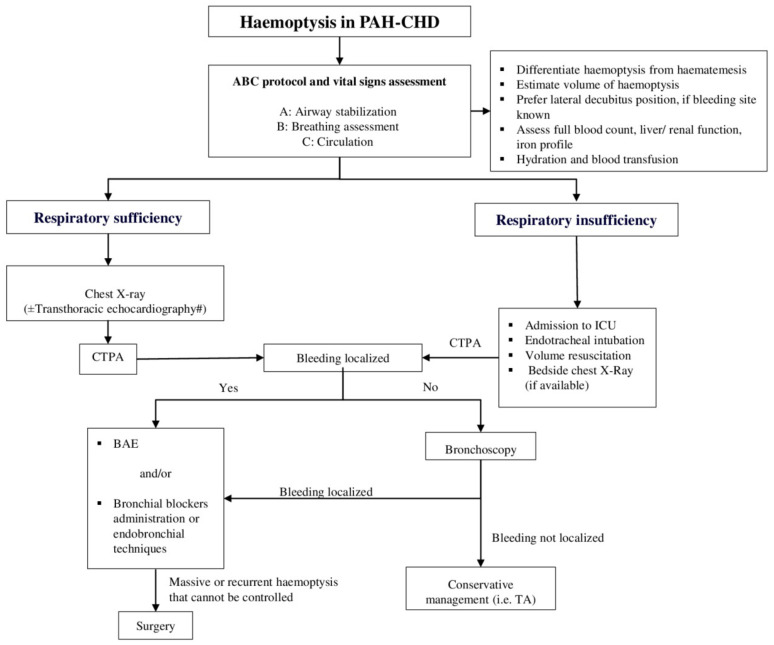
Proposed diagnostic and management algorithm of haemoptysis in patients with PAH-CHD. This algorithm has not been validated by a prospective study. # Cardiac magnetic resonance imaging may be additionally implemented to evaluate the intracardiac anatomy, abnormalities of the pulmonary vascular bed and the ventricular volumes. BAE: Bronchial artery embolization, CHD: Congenital heart disease, CTPA: Computerized tomography pulmonary angiography, ICU: Intensive care unit, PAH: Pulmonary arterial hypertension, TA: Tranexamic acid.

As haemoptysis constitutes a medical emergency, an immediate sophisticated therapeutic approach is required. Especially in case of massive haemoptysis, which represents the extreme degree of bleeding, treatment should be initiated concomitantly with the diagnostic evaluation. In Figure 2, we present a proposed diagnostic and management algorithm of haemoptysis in patients with PAH-CHD.

Applying the ABC protocol (airway, breathing, and circulation) and assessing the vital signs (arterial oxygen saturation, blood pressure, heart rate, respiratory rate) is the mainstay of care. In general terms, initial management principles focus on maintaining a patent airway, preventing hypoxia and securing hemodynamic stability, while thereafter quantification of haemoptysis and cessation of bleeding is also paramount. Further conservative or interventional measures to prevent recurrences once haemoptysis has been controlled and the patient is stabilized are warranted.

Securing sufficient oxygenation is the most important step in the initial management and can be achieved by the administration of high flow oxygen. On specific life-threatening occasions or if hypoxemia persists despite oxygen administration, airway maintenance requires endotracheal intubation. Several ways of reserving an airway using intubation in case of haemoptysis have been reported, including utilization of a single lumen endotracheal tube, a double-lumen endotracheal tube and a rigid bronchoscope. A large endotracheal tube with an internal diameter of about 8.5–9 mm is preferred in most cases, as it permits the transit of therapeutic flexible bronchoscopes or the use of bronchial blockers safely, without threatening the ventilation [53,55,56]. Blood suction and clot elicitation are performed efficiently in this way, assisting the stabilization of the patient [53].

Another significant step constitutes the positioning of the patient with the bleeding side down, if known from previous medical history or identified with imaging modalities, in order to prevent the expansion of bleeding to the rest of the intact regions of the lung [53,57]. In case the location of the haemorrhage is indeterminate, further diagnostic procedures, such as chest X-ray, CT scan or fiberoptic bronchoscopy, are required [48,53].

Prompt treatment of concomitant respiratory tract infections, reduction of physical activity and suppression of nonproductive cough are additionally supportive measures. Adequate hydration and blood transfusion in the presence of iron-replete anaemia (haemoglobin inadequate to oxygen saturation) are crucial. Furthermore, administration of platelets and/or fresh frozen plasma, vitamin K or coagulation factors may also be considered if haemoptysis persists [58].

### 5.2. Conservative Treatment: Tranexamic Acid

Tranexamic acid (TA), a synthetic derivative of the amino acid lysine, represents an antifibrinolytic agent used to control major bleedings. More specifically, it blocks the lysine-binding receptor sites on plasminogen and therefore inhibits competitively and reversibly the binding of plasminogen and plasmin to the lysine residues located at the fibrin. Consequently, plasmin, in turn, although formed, fails to bind to fibrin and activate its dissolution, leading to clot stabilization and impeding bleeding [59].

The stereo-isomer of TA and its antifibrinolytic action was first reported in 1964 by S. Okamoto et al. [60]. In the subsequent years, TA has been used to lessen blood loss in traumatic clinical settings [61], excessive bleeding after surgeries, and menorrhagia [59]. Regarding its effectiveness in haemoptysis, it has been studied only in few trials. Typically, TA is administered orally [62] or intravenously [63], yet inhaled [64,65] and endobronchial [66] forms have been recently discussed.

A recently published systematic review and meta-analysis demonstrated that the use of TA in patients with non-massive haemoptysis from any cause restricts the risk for further intervention procedures, moderates the bleeding volume and reduces the length of hospitalization by 1.62 days [63]. Another small randomized, controlled study of 66 patients with all-cause hemoptysis, although it failed to display a statistically significant superiority of TA over placebo, suggested a trend of improvement in frequency and quantity of haemoptysis [67]. Moreover, studies focused on the inhaled form of TA in patients with lung diseases concluded with encouraging results, whereas no significant adverse effects were noticed [64,65]. However, existing data on the use of TA as a therapeutic option in haemoptysis are derived from trials that included a limited number of patients and only non-massive cases of bleeding, while there is no evidence about its administration in PAH-CHD patients yet. Thereby, strong recommendations cannot be made, and further randomized clinical trials are required to ensure its efficacy in resolving haemoptysis, and especially in patients with PAH-CHD [68].

### 5.3. Endoscopic Management

As previously mentioned, the utility of bronchoscopy (with flexible or rigid bronchoscopes) is not only restricted in the diagnosis of haemoptysis, as it is expanded in treatment as well. In brief, it is performed to secure the airway, localize the haemorrhage in order to position the patient with the bleeding side down, intubate efficiently and administer bronchial blockers or implement other endobronchial techniques. Flexible bronchoscopy has merits with regard to airway visualization and access to distal airways [69]. In combination with CTPA, it should be recommended in non-life threatening cases of haemoptysis [42]. However, we should recognize the limitations of flexible bronchoscopy in managing massive life-threatening haemoptysis. Rigid bronchoscopy is safer and much more efficient at securing airway patency and preserving ventilation, allowing removal of large obstructing clots through its large working channel [54]. It also provides effective tamponade of the bleeding airway, and allows selective isolation of the nonaffected lung. In addition, bronchial blocking and local hemostasis are much easier to perform through the wider lumen of a rigid bronchoscope. Besides, a flexible fiber-optic bronchoscope can be introduced through the rigid scope to better evaluate the upper lobes and peripheral bronchi [70].

Repeated lavages with 50 mL aliquots of normal saline at 4 °C, by using rigid bronchoscopy, have been used successfully to terminate haemoptysis in patients with lung disease and prevent the need for emergency surgery [71,72]. Moreover, endobronchial administration of epinephrine and norepinephrine, arginine-vasopressin analogues (terlipressin or ornipressin) [73,74] and a topical hemostatic agent, called oxidized regenerated cellulose, have been reported to control haemoptysis attributed to lung pathologies [75]. Other therapeutic strategies of occluding the bleeding bronchus by using tamponade (with sterile surgical swabs) or balloon catheters have also been described in several studies over the years [57]. All of these bronchial blockers and techniques, primarily developed for lung diseases, could be potentially extrapolated to PAH-CHD patients. The rationale, nevertheless, includes concerns regarding their effectiveness in this specific patient group, as reassuring data from large controlled trials are lacking.

### 5.4. Bronchial Artery Embolization

**Figure 3:**
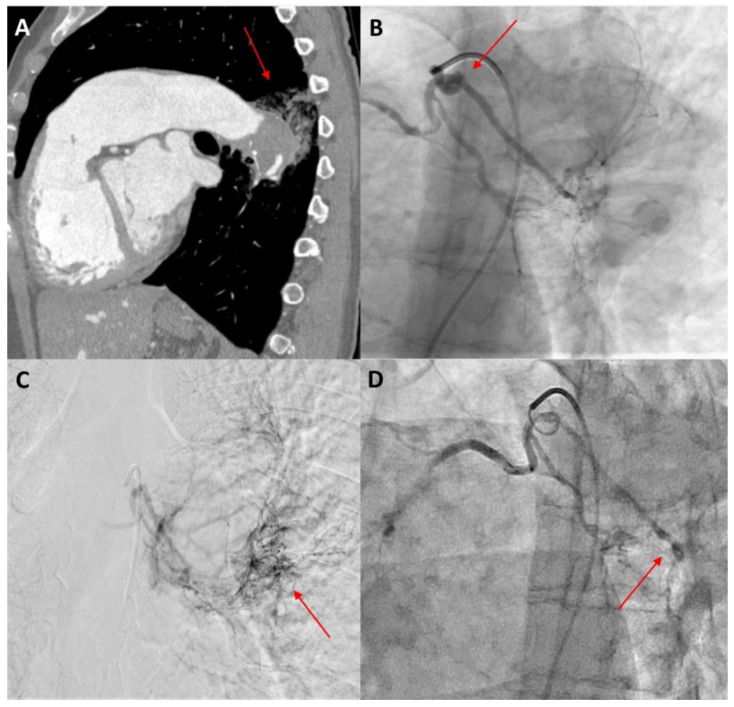
A 40-year-old female patient with Eisenmenger syndrome on the background of a large secundum ASD presenting with recurrent episodes of haemoptysis. (**A**) Sagittal view of thorax computed tomography after intravenous contrast medium injection, showing aneurysmatic dilatation of the left pulmonary artery with intraluminal thrombus and adjacent post-bleeding infiltrations in the lung parenchyma (red arrow). (**B**) Catheterization of the bronchial arteries with a 5 Fr Cobra catheter, showing the tortuous origin of the left bronchial artery (red arrow). (**C**) Angiographic image in a delayed parenchymal phase, showing abnormal imaging of the left posterior lung parenchyma adjacent to the descending aorta, corresponding to the area of bleeding (red arrow). (**D**) Angiographic image after embolization with 100–300 μ particles through a 2.6 Fr microcatheter. The left bronchial artery could not be selectively catheterized, so injection of particles was performed in both bronchial arteries until stasis was achieved, especially in the left side (red arrow).

Bronchial artery embolization (BAE) is a minimally invasive and efficient technique in managing massive and recurrent haemoptysis as a single therapy or in combination with conservative medication or surgery [13,62]. BAE, first introduced by Remy et al. in 1974 [76], is performed in view of lowering the perfusion pressure by occluding the systemic arterial inflow of the culprit vessels [25]. As an overall success rate of 70–99% has been documented over the years, it is now considered as a first-line therapy to control haemoptysis in many cases [77]. Before the procedure, an arteriogram should be performed to visualize the responsible bronchial arteries and nonbronchial collateral arteries that will be the target of embolization [13,78] (Figure 3).

To date, corroborating evidence on the role of BAE in patients with PAH-CHD is limited and comes mostly from case reports and small cohorts. Successful and uncomplicated embolization of the bronchial arteries or the systemic-to-pulmonary collaterals has been described in several single cases of patients with ES [36,79]. Two observational studies that included 12 and 8 PAH-CHD patients presenting with haemoptysis (of which four patients had ES) confirmed the safety and efficacy of BAE in this specific population [78,80]. The estimated recurrence rate after BAE has been noted as being remarkably high in the latter cohort, rising to as much as 50% [80]. Generally, deficient embolization of the culprit vessels is responsible for early re-bleeding, whereas late-term recurrence may result from recanalization or further hypervascularization leading to the formation of new collaterals [13,81]. Combined therapy with BAE and TA may assist in achieving lower relapsing rates following BAE [62].

Complications associated with BAE are usually temporary and attributed to the accidental occlusion of branches of bronchial arteries that supply the esophagus, diaphragmatic and mediastinal visceral pleura, and spinal cord [13]. Transient chest pain and dysphagia are the most commonly encountered complications associated with BAE, occurring in 24–90% and 1–20% of cases, respectively [81]. The most threatening complication is transverse myelitis owing to spinal cord ischemia following the inattentive embolization of the anterior spinal artery, estimated in 1.4–6.5% of cases [81]. Moreover, esophageal ischemia, aortic or bronchial necrosis, distal non-target organ infarction (regression of embolic material into the aorta that may lead to stroke and/or cortical blindness) are rarely described complications [25,77,81].

### 5.5. Surgical Management

Since significant progress in bronchoscope and BAE techniques has been made, the surgical approach (lung resection) is no longer the treatment of choice in controlling massive and recurrent haemoptysis in PAH-CHD, as it entails a high risk of mortality, and is indicated only in case conservative treatment and BAE prove unsuccessful [53,80,81]. Rescue (heart-) lung transplantation could presumably be a permanent solution in patients with ES and massive or recurrent episodes of haemoptysis. Nevertheless, it is infrequently attempted, in an emergency setting, because of the scarcity of lung transplants and the high risk of transplant rejection [82].

### 5.6. Anticoagulation

The need for oral anticoagulant treatment for primary or secondary prevention in PAH-CHD remains controversial, and individually-based decisions should be taken in expert centres [2,83]. The increased thrombotic risk, imputed to biventricular dysfunction, right atrial blood stasis, dilated pulmonary arteries, atherosclerosis, endothelial dysfunction, the presence of thrombogenic material (e.g., conduits), and arrhythmias [2,10,68] could support the rationale for oral anticoagulation [84,85]. Current guidelines recommend its use only in patients with low bleeding risk and concomitant atrial arrhythmias, PA thrombus or other prior thromboembolic events. Consequently, routine use of anticoagulation therapy for primary prevention is discouraged due to lack of supportive evidence [68].

Typically, Vitamin-K antagonists (VKA) are used for thromboprophylaxis in PAH-CHD, whereas aspirin is sometimes used empirically in view of the notion that antiplatelets cause fewer bleeding adverse events in cyanotic patients [68,84]. Moreover, the extrapolation of VKA replacement with direct oral anticoagulants in CHD is questionable because of insufficient supporting data [68,86,87].

In patients presenting with active haemoptysis, provisional cessation of existing anticoagulation or antiplatelet treatment seems reasonable [88]. Prospective data from two single centres studies revealed that 13% to 23.6% of the included PAH-CHD patients with haemoptysis received anticoagulants before the episode [10,80]. In the absence of guidelines and randomized clinical trials enlightening on the issue of reestablishment of anticoagulation therapy following haemoptysis resolution, each decision should be based on a case-by-case consideration, taking into account both bleeding and thromboembolic risk [68]. Cessation of anticoagulation therapy is the current clinical practice applied in the majority of tertiary centres. More studies are warranted to shed light on the impact of anticoagulation on haemoptysis recurrence and guide everyday clinical practice.

## 6. Prognosis

Regarding haemoptysis recurrence and its effect on patients’ quality of life, limited data exist [89]. In ES, estimated relapse rates between 17% and 20% have been reported [7,49] (Table 1). Nevertheless, there is no documented evidence on hospitalization rates due to multiple haemoptysis episodes.

A legitimate argument has been raised about the impact of haemoptysis on mortality in PAH-CHD. In Paul Wood’s series, 29% of deaths in patients with ES were attributed to haemoptysis [35]. Saha et al. described haemoptysis as contributing to death in 15% of cases [7], whereas subsequent studies have reported a mortality rate of 3% to 11.4% in patients with PAH-CHD presenting with haemoptysis (Table 1) [8,9,12]. However, the current literature establishes no impact on survival; hence haemoptysis should not be considered as a fatal clinical manifestation or a deteriorating prognostic factor in this population [5,6,8,9]. The reported decrease of fatal haemoptysis in PAH-CHD over the last decades may be partly explained by the significant steps forward in clinical understanding and medical management of these episodes, especially in the context of recent advances in interventional techniques as well as the more effective medical management of pulmonary vascular disease.

Nevertheless, one could argue that haemoptysis and especially recurrent episodes may reflect disease progression that requires the escalation of PAH treatment and possibly an earlier patient referral for transplantation assessment [90].

## 7. Conclusions

Haemoptysis in PAH-CHD requires a multidisciplinary, personalized decision-making approach in specialized centres. Although infrequently fatal, haemoptysis is associated with increased morbidity burden, multiple psychological effects and a poor quality of life. BAE attempted to alter the management landscape; nevertheless, there is still little information on its definite benefits for patients with PAH-CHD. Further studies and prospective registries are warranted to guide well-documented therapies with the potential for optimal results that will provide insight into the treatment gaps.

## Figures and Tables

**Table 1:** Observational studies evaluating incidence, management and recurrence of haemoptysis in patients with PAH-CHD.

Author (Year)	Study Type	Study Population	Age (Years)	Follow-Up	Haemoptysis N (%)	BAE N (%)	Haemoptysis Related Mortality N (%)	Recurrence of Haemoptysis N (%)
P. Wood (1958)	Prospective single centre	ES n = 127	ES-PDA: 19	11 years	N/A	N/A	12 (29)	N/A
			ES-VSD: 22					
			ES-ASD: 35					
A. Saha (1994)	Retrospective study	ES n = 201	19.23 ±12.62	54.6 ± 54.47 months	34 (16.9)	N/A	3 (15)	31 (17)
L. Daliento (1998)	Retrospective multicentre study	ES n = 188	25 (IQR 17–34)	31 (IQR 22.5–43.0) years	38 (20.2)	N/A	7 (3.7)	N/A
W. J. Cantor (1999)	Retrospective single centre study	ES n = 109	28.6 ± 10.8	Median: 6.3 years	34 (31)	N/A	1 (3)	N/A
C. S. Broberg (2007)	Prospective cross-sectional single-centre study	ES n = 55	N/A	-	27 (49) PA thrombosis N = 11 (20)	N/A	N/A	N/A
J. Cantu (2012)	Retrospective single-centre study	PAH-CHD n = 8 (with hemoptysis) ES n = 4	38.1 ± 14.2	36 (IQR 3.6–59.1) months	8 (100%)	6 (75%)	0	4 (50) of PAH-CHD ^#^
M. J. Schuuring (2015)	Prospective single-centre study	PAH-CHD n = 91	42 ± 14	4.7 (R 0.1–7.9) years	5 (5.5)	N/A	1 (4.1)	N/A
A. S. Jensen (2015)	Prospective, longitudinal, single-centre study	ES n = 48	40 ± 14	8–10 years	N/A	N/A	N/A	2 (20)
S. Hascoet (2017)	Retrospective, observational, nationwide, multicentre cohort study	ES n = 340	26.5 (IQR 11.9–39.7)	5.5 (IQR 3.0–9.1) years	43 (12.6)	N/A	N/A	N/A
C. M. S. Hjortshøj (2017)	Retrospective, observational, multicentre study	ES n = 1546	38.7 ± 15.4	6.1 (IQR 2.1–21.5) years	92 (6)	N/A	17 (3)	N/A
E. Rasciti (2017)	Prospective single-centre study	PH n = 21 PAH-CHD n = 12 (Haemoptysis and BAE)	38.96 ± 14.33	At 30- and 90-days post BAE	12 (100)	12 (100)	N/A	NA
D. Ntiloudi (2019)	Prospective, nationwide, multicentre study	PAH-CHD n = 65	46.1 ± 14.4	3 (IQR 1–6) years	2 (3.1)	N/A	N/A	N/A

^#^ 3 pts treated with BAE and 1 pt. treated medically. ASD: atrial septal defect, BAE: Bronchial artery embolization, ES: Eisenmenger syndrome, IQR: interquartile range, PAH-CHD: Pulmonary arterial hypertension associated with congenital heart disease, PA: pulmonary artery, PDA: patent ductus arteriosus, R: range, VSD: ventricular septal defect.
